# Insights into the Complex Formed by Matrix Metalloproteinase-2 and Alloxan Inhibitors: Molecular Dynamics Simulations and Free Energy Calculations

**DOI:** 10.1371/journal.pone.0025597

**Published:** 2011-10-05

**Authors:** Ilenia Giangreco, Gianluca Lattanzi, Orazio Nicolotti, Marco Catto, Antonio Laghezza, Francesco Leonetti, Angela Stefanachi, Angelo Carotti

**Affiliations:** 1 Dipartimento Farmaco-Chimico, University of Bari “Aldo Moro”, Bari, Italy; 2 Dipartimento di Scienze Mediche di Base, University of Bari “Aldo Moro”, Bari, Italy; 3 Centro Ricerche Tires, University of Bari “Aldo Moro”, Bari, Italy; 4 Istituto Nazionale di Fisica Nucleare, University of Bari “Aldo Moro”, Bari, Italy; Russian Academy of Sciences, Institute for Biological Instrumentation, Russian Federation

## Abstract

Matrix metalloproteinases (MMP) are well-known biological targets implicated in tumour progression, homeostatic regulation, innate immunity, impaired delivery of pro-apoptotic ligands, and the release and cleavage of cell-surface receptors. Hence, the development of potent and selective inhibitors targeting these enzymes continues to be eagerly sought. In this paper, a number of alloxan-based compounds, initially conceived to bias other therapeutically relevant enzymes, were rationally modified and successfully repurposed to inhibit MMP-2 (also named gelatinase A) in the nanomolar range. Importantly, the alloxan core makes its debut as zinc binding group since it ensures a stable tetrahedral coordination of the catalytic zinc ion in concert with the three histidines of the HExxHxxGxxH metzincin signature motif, further stabilized by a hydrogen bond with the glutamate residue belonging to the same motif. The molecular decoration of the alloxan core with a biphenyl privileged structure allowed to sample the deep S_1_′ specificity pocket of MMP-2 and to relate the high affinity towards this enzyme with the chance of forming a hydrogen bond network with the backbone of Leu116 and Asn147 and the side chains of Tyr144, Thr145 and Arg149 at the bottom of the pocket. The effect of even slight structural changes in determining the interaction at the S_1_′ subsite of MMP-2 as well as the nature and strength of the binding is elucidated via molecular dynamics simulations and free energy calculations. Among the herein presented compounds, the highest affinity (pIC_50_ = 7.06) is found for **BAM**, a compound exhibiting also selectivity (>20) towards MMP-2, as compared to MMP-9, the other member of the gelatinases.

## Introduction

**Figure 1:**
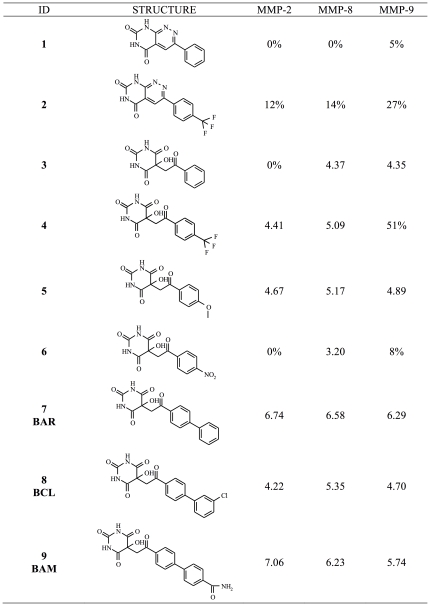
Biological data of alloxan-based compounds 1–9. We report the pIC_50_ or % of inhibition at 100 µM.

Matrix metalloproteinases (MMPs) are a family of zinc- and calcium-dependent endopeptidases involved in the degradation of the extracellular matrix (ECM) [1]. They play a key role in tissue turnover and remodelling and their over-expression is a hallmark of various inflammatory, malignant, and degenerative diseases [2]–[4]. Such evidence has led scientists, in both academia and industry, to make considerable efforts in the attempt to develop new MMP inhibitors (MMPIs) to contrast dysregulation of such important enzymes [5]. At present, several potent and orally available broad spectrum MMPIs have been discovered. However, the toxicity and dose-limiting efficacy emerged in clinical trials, supposedly due to non-specific inhibition, have clearly stressed the need for more selective compounds discriminating among different members of the MMP family [6], [7]. In view of this, great efforts have been addressed to selectively target MMP-2 [8], better known as gelatinase A, that plays a central role in angiogenesis given its catalytic action in the hydrolysis of collagen type IV, the main component of the basement membrane, as well as of interstitial collagens like type I [9]. Besides, the expression of MMP-2 is related to the appearance of many different human tumours and inflammatory diseases. Likewise other MMPs, MMP-2 contains a common sequence motif, HExGHxxGxxH that is characterised by three histidine residues coordinating the catalytic zinc ion, and also shares five-stranded-β-sheets (one antiparallel and four parallel) as well as three α-helices in the zinc-based endopeptidase fold. Nevertheless, MMP-2 has an own typical three-dimensional structure with a catalytic domain incorporating three fibronectin type-II-like modules that mediate interaction with substrates such as gelatin, laminin and collagens [10], [11]. The active site is constituted by a cavity traversing the entire enzyme and structured in a number of specific subsites interacting with physiological substrates and targeted by natural or synthetic inhibitors. A relevant role to ensure a potent and selective binding [12] is exerted by the zinc metal ion acting as an anchoring site for many zinc-binding groups (ZBGs) [13]. Besides simple functional groups (e.g., hydroxamic and carboxylic acids, thiols and sulfonamides), a number of higher structured molecular fragments were successfully examined as better selective ZBGs (e.g., barbiturates, hydroxypyrones and hydroxypyridones) being the chelating action towards the zinc metal ion further reinforced by the specific occurrence of a stronger interaction in terms of hydrogen-bond network and van der Waals (vdW) contacts with the protein, resulting in a consistent gain of molecular affinity and selectivity [14]. In this respect, a wealth of information for identifying novel chelating scaffolds was found in a local academic collection of about 2,000 diverse and good quality compounds prepared by our research group over the last 25 years [15], [16], [17]. It represents the front-line of our investigations having already being successful for the discovery of promising anticancer lead compounds in a three years-project CGRID funded by the European Union [18]. Our analysis was thus directed towards compounds having functional groups or even larger molecular substructures acting as potential ZBGs. In this respect, our attention was engaged by a number of reaction intermediates contained within the library that are based on the alloxan core originally designed for the preparation of a panel of condensed pyridazines acting as Monoamine Oxidase-B inhibitors (i.e., **1** and **2** in Figure 1) [19]. A few alloxan derivatives were thus prepared as investigational models to study the *in vitro* inhibition of gelatinase MMP-2. As a complement, further biological assays were lately executed on MMP-9, the other gelatinase, and on collagenase MMP-8. Satisfactorily, valuable binding affinities and molecular selectivity were observed. In the present work, our attention is directed to shed light on the molecular determinants responsible for the binding affinity towards MMP-2. To this end, the highest (i.e., **9** in Figure 1, named **BAM** in the following) and lowest (i.e., **8** in Figure 1, named **BCL** in the following) active alloxan derivatives were subjected to molecular dynamics (MD) [20] and free energy calculations [21] to explain quantitatively the gap of observed binding energies in comparison to the unsubstituted derivative (i.e., **7** in Figure 1, named **BAR** in the following) that was used as a reference.

## Materials and Methods

### Setup of the systems and docking calculations

A chemical library of about 2,000 compounds was investigated with the aim of finding potential ZBGs. It is a private academic repository collecting all the molecules synthesized in our research group over the last 25 years. Such molecules have a typical drug-like profile being their design biased towards a number of biological targets of our interest, e.g. monoamineoxidases (MAOs), cholinesterases, aromatases, topoisomerases, serine and cysteine proteases, diverse G protein coupled receptors and benzodiazepine receptor. All these structures along with a number of biological and physicochemical properties have been stored in a local electronic library. After retrieving chemical structures in a 2D format, reliable molecular conformations were automatically generated by CORINA, a 3D structure generator tool, [22] and passed to GOLD [23] for docking simulations into the MMP-2 binding site. Initial coordinates for the protein were taken from the first NMR model (PDB entry: 1HOV), which corresponds to the solution structure of a catalytic domain of MMP-2 complexed with a hydroxamic acid inhibitor (i.e., SC-74020) [24].

GOLD was set to generate 10 docking poses for each molecule. Residues within an active radius of 12 Å centred on the catalytic Zn^2+^ ion were explicitly accounted in docking sampling. GOLD was flagged for the automated determination of the coordination geometry around the zinc metal ion. More specifically, virtual coordination points were added at locations where GOLD was missing a coordination site and these coordination points were used as fitting points that could bind to acceptors. In order to determine the coordination geometry, GOLD performs a permuted superimposition of coordination geometry templates onto the coordinating atoms found in the protein. Coordination fitting points were thus generated using the template that gave the best fit in terms of RMSD from the target. The tetrahedral and trigonal bipyramidal coordination geometries were manually specified to allow the docking software to prioritize the Zn metal ion. A visual inspection of poses resulting from alloxan-based structures disclosed conformations that matched the structural requisites for a stable coordination with the metal as well as a suitable orientation of the biphenyl fragment into the S_1_′ subsite. Such evidence increased our confidence in pursuing the molecular development of these hits. As a proof of this, the reference compound **BAR** was tested resulting in an inhibitory affinity in the low nanomolar range. In continuing work, further docking simulations were performed setting a deeper sampling of the genetic algorithm and increasing the number of solutions per molecule up to 50. To obtain an initial structure for the complex formed between the catalytic domain of the MMP-2 enzyme and the alloxan derivatives, results coming from automatic docking calculations were analysed along with the valuable structural information derived from PDB [25] accounting for complexes between barbiturate ligands and MMPs. The only difference between the alloxan and 5,5-disubstituted barbituric acid derivatives is the presence of a 5-hydroxyl group in the former. However, such a little structural variation makes the two compounds strongly diverse in terms of acidity and of possible tautomeric equilibria.

**Figure 2:**
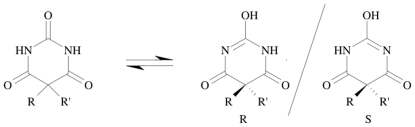
Keto-enol tautomerism referred to the chemical equilibrium between the keto and enol form established by the alloxan-like structure as described on the left-hand and right-hand side, respectively.

At the time of writing, three PDB structures of MMPs complexed with barbiturate inhibitors were available [26], [27]. All of them showed preferred tautomers with the N1 atom incorporated into the metal coordination sphere having a tetrahedral geometry, while the enolic group established a hydrogen bond (HB) with the charged Glu121 (numbering as in 1HOV) in the active site. As a result, the enolic form of the barbiturate is supposed to be favoured by the protein matrix over the tautomeric keto form (that is instead prevalent in solution) [28] (Figure 2). With this in mind, a docking pose complying with a high score value and a ligand conformation resembling crystallographic data was used as the initial structure for MD simulations.

### MD simulations

**Figure 3:**
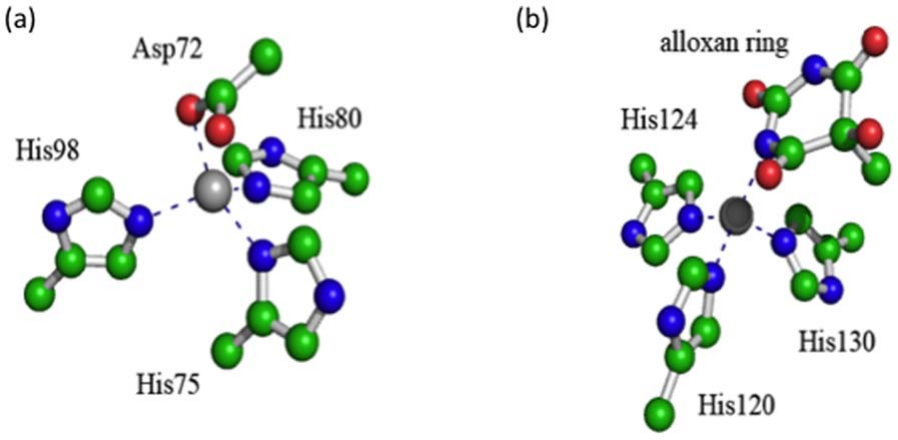
Bonded representation of the coordination at the structural (a) and catalytic (b) Zn^2**+**^ ions of MMP-2.

The X-ray structure of MMP-2 (PDB code 1HOV) [24] was retrieved and, after the removal of the inhibitor, complexed with the three selected alloxan derivatives (i.e., **BAR**, **BCL** and **BAM**) by inserting docked ligands into the protein binding site. The protonation states of the histidine residues were adjusted according to the hydrogen bond network, while basic amino functional groups were protonated, aromatic amino functional groups were left uncharged and carboxylic groups were considered to be deprotonated. The non-bonded representation proposed by Aqvist was employed for the calcium ions, here considered with a 2+ charge [29]. A bonded representation for the zinc ions was constructed by placing explicit bonds between the metal and the coordinating atoms, after separately parametrizing each of them by Hartree-Fock calculations at the HF/6-31G* level of theory. In particular, for the structural Zn^2+^ ion (Figure 3a), the adopted bonded representation corresponds to one in which the metal ion is linked to the His98-Nε, His70-Nε, His75-Nδ and the Asp72-O atoms by explicit molecular mechanics bonds. The catalytic Zn^2+^ ion (Figure 3b) was explicitly linked to the His120-Nε, His124-Nε, His130-Nε atoms and the N atom of the alloxan ring.

The potential energy surface (PES) of the two Zn moieties was analyzed by using the Gaussian package [30]. A single point energy calculation, with geometry fixed, was first performed and used as a reference for the subsequent geometry optimization. Then, a further single point calculation was performed on the obtained optimal geometry. The results were finally used to derive the RESP (Restrained Electrostatic Potential) charges and complete the parametrization of the two Zn moieties [31].

The analyzed ligands were sketched and parametrized with GAFF atom types [32]. Each structure was immersed in a cubic TIP3P water box that extended 18 Å from the protein atoms and neutralized by addition of Na^+^ counter ions using the AMBER Leap module [33]. This led to a simulation system of 54,630 atoms in a box of 82.84×88.37×80.06 Å^3^. The parm03 version of the all-atom AMBER force field was used to model the system [34]. The solvent molecules were initially relaxed by energy minimization and subsequent 30 ps of MD. The full systems were further minimized to remove bad contacts in the initial geometry and heated gradually to 310 K during 600 ps of MD. The SHAKE algorithm was employed to constrain all R–H bonds. Periodic boundary conditions were applied in all directions. The cut-off for non-bonded interactions was set to 12 Å. Particle-Mesh-Ewald (PME) was employed to include the contributions of long-range interactions with 64 nodes in all directions [35]. Pressure (1 atm) and temperature (310 K) were controlled during the MD simulations by the Langevin method [36]. NAMD [37] was used to compute 5 ns of equilibration trajectory for each model with a time step of 1.5 fs. Coordinates were saved for analysis every 0.6 ps.

### Free energy calculations

**Figure 4:**
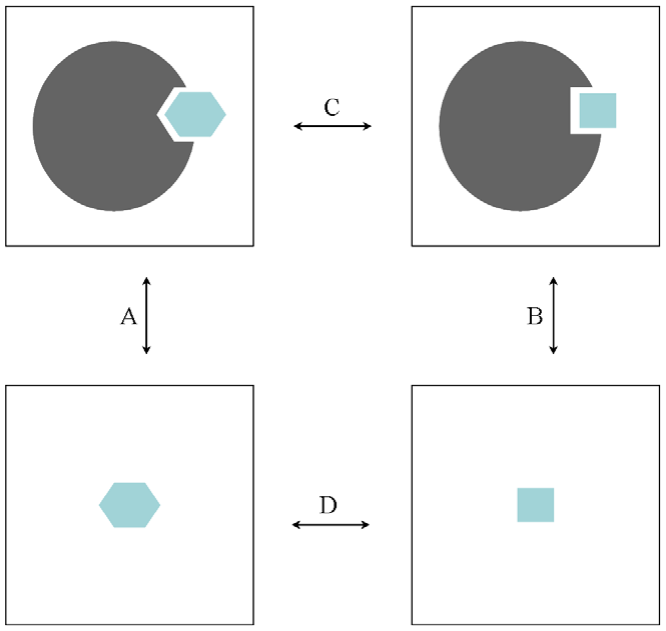
Thermodynamic cycle. Events A and B represent the binding of two different ligands to a protein, events C and D indicate the conversion from one ligand to the other in the bound and hydrated states, respectively. The free energy differences between the processes A and C can be obtained calculating the free energy differences between B and D.

Thermodynamic integration (TI) computes the free energy difference between two states A and B by coupling them via a parameter lambda, which serves as an additional, non-spatial coordinate. This lambda formalism allows the free energy difference between the states to be computed as:
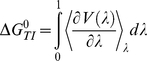
(1)The thermodynamic cycle showed in Figure 4 allows the comparison of results from a series of TI calculations with physical observables.

Processes A and B represent the binding of two different ligands to a protein, while processes C and D are transformations from one ligand to the other while it is bound to the protein (C) or simply solvated in water (D). Since ΔG_C_−ΔG_D_ = ΔG_A_−ΔG_B_, TI calculations can be used to compute relative binding free energies, making them useful tools in drug design or lead optimization applications. In equation 1, V(λ) is the λ-coupled potential function that corresponds to V(A) for λ = 0 and V(B) for λ = 1. The integration was carried out over the average of the λ derivative of the coupled potential function at given λ values. Since this integration can only rarely be performed analytically, an integration scheme was used in which simulations at different discrete λ points were performed and the value of the integral was numerically evaluated by interpolation. A benefit of TI calculations is that several independent MD simulations at fixed λ values can be performed independently, allowing for efficient parallelization. Moreover, additional λ points can be added at any stage to improve accuracy.

In this work, free energy calculations were used to calculate the relative binding free energies of the three alloxan inhibitors **BAR**, **BCL** and **BAM**. Free energies were computed by using the thermodynamic integration tools of the SANDER routine implemented in AMBER 10.0, with modified vdW interactions (softcore potential) to ensure smooth free energy curves [38].

Each calculation consisted in two different ligand transformations: i) molecule 1 to molecule 2 in water and ii) bound to MMP-2. For consistency, both transformations were further broken up into three sub-steps each: step 1 consists in the removal of the partial charge on the atoms that would be substituted or deleted from molecule 1 (for instance, the hydrogen atom of **BAR**); in step 2, atomic species are substituted with no charges, hence resulting in the calculation of the contribution of the vdW interaction to the free energy difference (in our case, the hydrogen atom on **BAR** was deleted while simultaneously inserting the chlorine atom on **BCL** or the carboxamido group on **BAM**). Finally (step 3), the atomic charges on the substituted groups are switched on. This procedure is required to avoid instabilities due to the presence of a non-zero charge on an atom whose vdW interactions get progressively weaker. Softcore potentials were used for all the steps 2 in our transformations to prevent the free energy divergence caused by the zero-point singularity. In particular, the van der Waals interactions were modified as follows [39]:
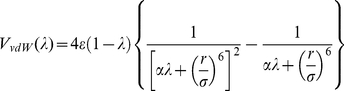
(2)This means that the Lennard-Jones interactions include the parameter λ so that there are no singularities when λ approaches the value 0 or 1.

**Figure 5:**
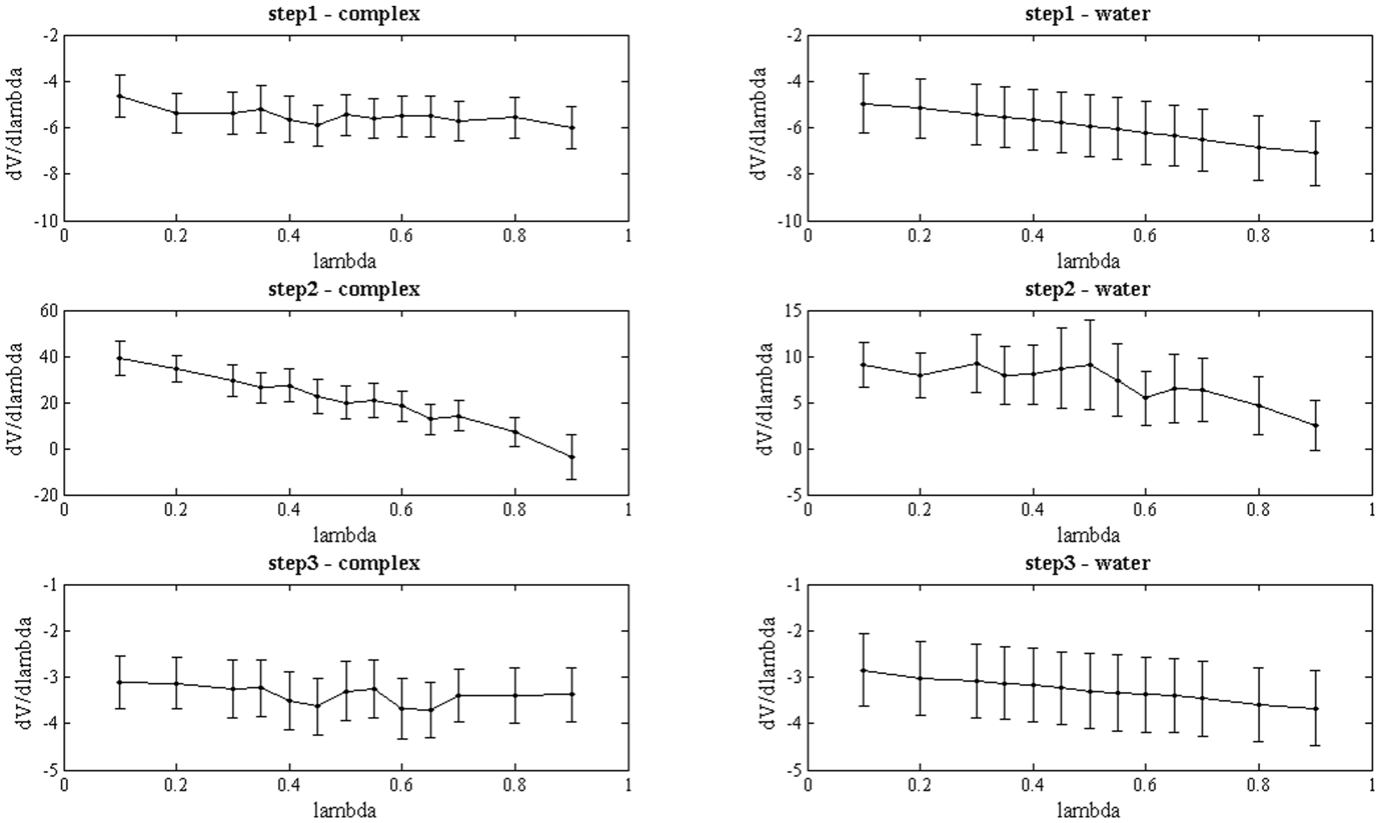
Thermodynamic integration for the case BAR to BCL. The plot shows the dV/dλ curves assembled from 13 lambda points. Transformations in water are represented as well as those in the protein for the three simulation steps.

Each sub-step at a definite value of λ consisted in 500 steps of steepest descent minimization, followed by 50 ps of density equilibration and 200 ps of NPT production MD to collect dV/dλ data. A time step of 2 fs was used together with the SHAKE algorithm, with the same parameters as for the equilibration. As an example, Figure 5 shows the forward transformation of **BAR** into **BCL**. Errors were estimated as reported in ref. [39] and the total integration error was calculated by using error propagation on the sums, differences and interpolated values.

### Chemistry

**Figure 6:**
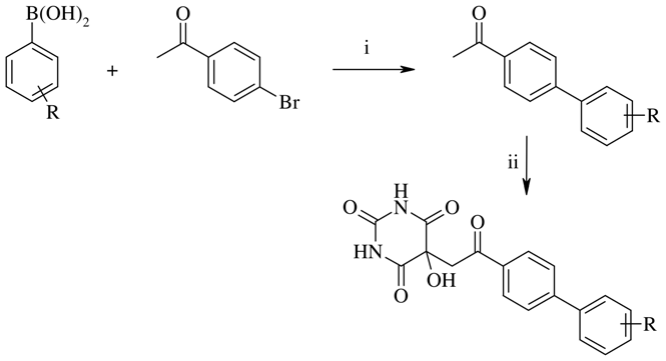
Synopsis of the synthesis of compounds 3–9 in **Figure 1**. Reagents and conditions: (i) Na_2_CO_3_, (PPh_3_)_2_PdCl_2_, water/dioxane, microwave heating; (ii) alloxan monohydrate, acetic acid, reflux.

Compounds in Figure 1 were prepared via aldol condensation of alloxan and suitable methylketones as already described [19]. Substituted biphenyl methyl ketones for **BCL** and **BAM** were prepared via Suzuki-Miyaura microwave assisted synthesis from 4-bromoacetophenone and corresponding arylboronic acids (see Figure 6).

### MMP Inhibition assays

Inhibitory activities were determined by a fluorimetric assay, where a pro-fluorescing peptide was used as substrate of MMP, and the fluorogenic activity of its cleavage product was measured after co-incubation with test compounds.

## Results and Discussion

**Figure 7:**
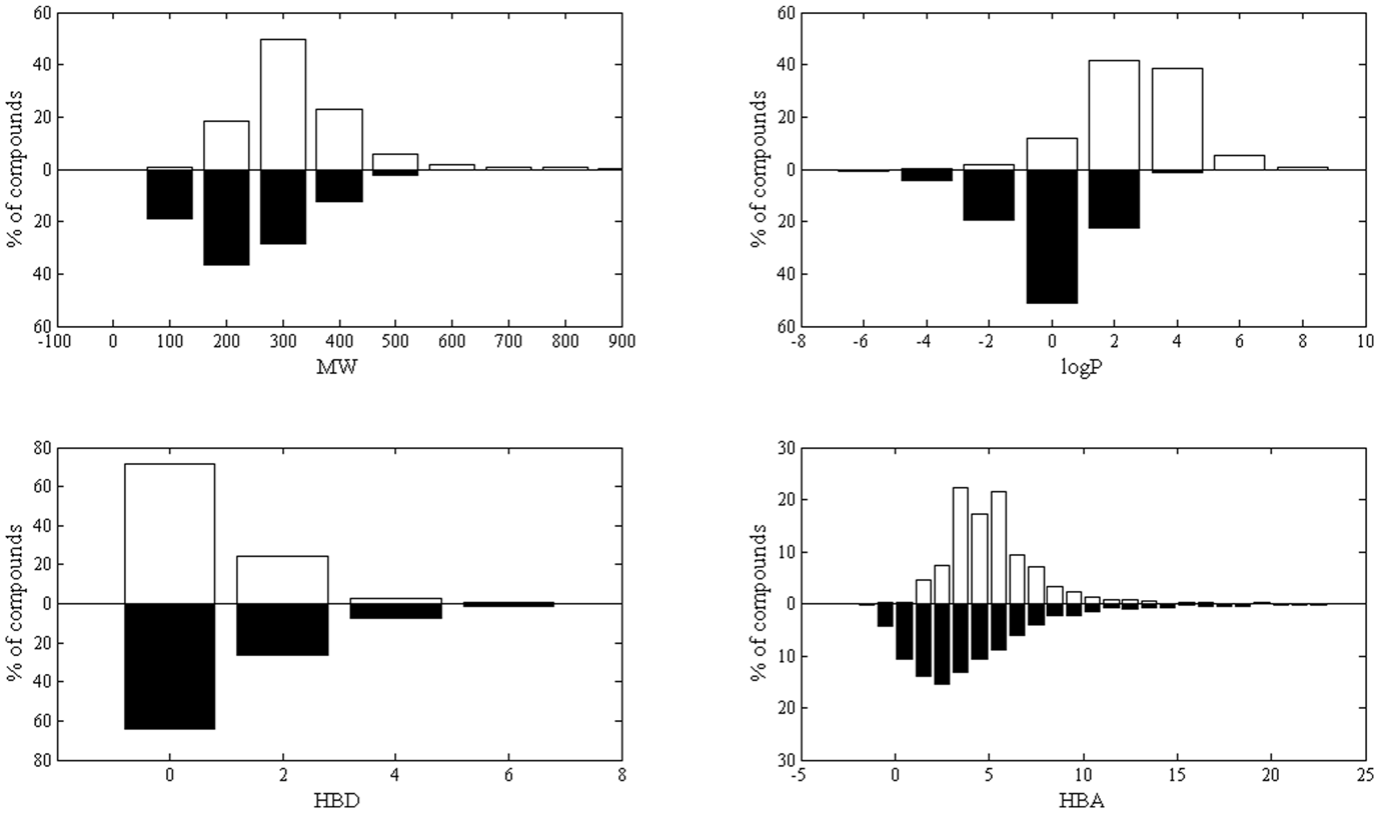
The physical property profiles of our academic collection (in black) are compared with property profiles found in DrugBank (in white). The y-axis represents the percentage of compounds, the x-axis represents the molecular weights (MW), logP, hydrogen bond donor (HBD) and hydrogen bond acceptors (HBA) profiles.

A chemical library of about 2,000 rationally designed compounds previously synthesized in our research group was used as starting material for this work. It is evident that the quality of chemical collection is increased if compounds contained within the library have drug-like properties. However, it is difficult to define clearly the concept of druglikeness in terms of the exact characteristics that a molecule should have in order to be viable as a drug. There are some general criteria that can be applied in compound acquisition programs to filter out undesirable compounds. A milestone of filtering techniques is the well known Rule-of-five developed by Lipinski [40]. The rule is based on easy-to-calculate properties that are designed to identify compounds that are likely to exhibit poor intestinal absorption. In this respect, physical properties relevant to assess druglikeness (i.e., molecular weight, logP, number of hydrogen bond donor atoms and number of hydrogen bond acceptor atoms) were calculated for each compound of our library. As shown in Figure 7, the likelihood of our library to exhibit activity in any therapeutic area was estimated by comparing its bioactive profiles with those derived from DrugBank [41], a freely available database containing FDA-approved small molecule drugs, FDA-approved biotech drugs, nutraceuticals and experimental drugs.

**Figure 8:**
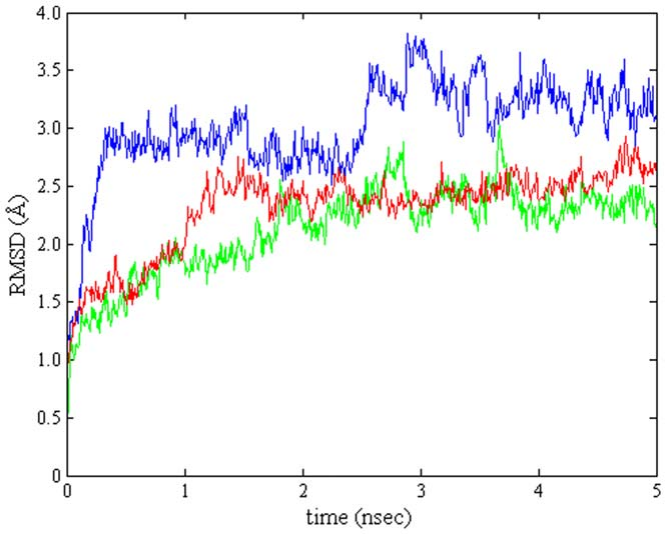
RMSD of the C-alpha carbon atoms of MMP-2 (PDB code: 1HOV) for the equilibration trajectories. The protein complex with **BAR** (green line), **BCL** (red line) and **BAM** (blue line) is stable within the time frame of equilibration.

Our local academic collection was thus interrogated via sub-structural similarity searches of known ZBGs. Interestingly, besides the presence of a few canonical ZBGs (i.e., thiols or carboxylic acids), four alloxan-based structures (i.e., derivatives of the 2,4,6-trioxopyrimidines) were also fished as potential hits. The rationale behind such selection was that some 5,5-disubstituted-2,4,6-trioxopyrimidines were already known as MMPIs [42], [43], [44]. Actually, alloxan-based compounds were stored as chemical intermediates for the rational design of pyrimidopyridazine derivatives biasing the monoamineoxidases (MAO-A and MAO-B) enzymes as potential therapeutics for Parkinson's disease [19]. However, our attention was engaged by the presence of the 5-OH group and by its potential role in presumably determining both metal coordination as well as an appropriate hydrophilic-lipophilic balance in such hydrophobic molecules. In this respect, the bioactive potential of alloxan-based compounds available from our collection was challenged by docking calculations with the result that valuable scoring and posing values towards MMP-2 enzymes were found. These initial observations increased our confidence in the potential role of these compounds as MMP-2 inhibitors. Interestingly, preliminary biological experimental data awarded a number of these selected derivatives with inhibition measures in the high micromolar range. Such selected alloxan compounds were thus improved through a knowledge-based molecular design. Basically, the phenyl moiety of compounds **3** in Figure 1 was augmented to biphenyl (i.e., **BAR** in Figure 1), being such a molecular fragment a privileged structure for targeting the S_1_′ subsite of MMPs. The need of capturing selective interactions into the S_1_′ subsite prompted us to consider further molecular optimizations. In this respect, the distal aromatic ring of the biphenyl moiety was thus further decorated at the *meta* and *para* positions by inserting two properly selected substituents with easy commercial and chemical access as well as different Hammett σ and Hansch π constants [45]. These compounds could be promising in the field of MMP inhibition, considering that compounds under clinical investigation are hydroxamic acid derivatives with similar inhibition patterns (e.g., BB-2516, Ro 32-3555) [46], [47]. On the other hand, the alloxan ring, a ZBG other than the hydroxamic group, makes these compounds higher desirable as they have better drug-like properties and are presumably less toxic *in vivo*. In this respect, **BAR** was used as lead compound for the next substitution on the biphenyl ring. In this work, we considered a small series of derivatives differing for type and position of substitution. Inhibition data showed that the *meta*-chloro substituted biphenyl derivative is less active than the *para*-carboxamido analogue, which resulted as the most active and selective MMP-2 inhibitor. Docking calculations assisted and drove the design and synthesis of this series of compounds. However, as reported in literature, established scoring functions fail to predict binding affinity of slightly different molecules as in the case of our series comprising compounds varying only for mono-substitutions in the distal aromatic ring of the biphenyl fragment. As shown in Table 1, score values obtained after docking compounds **BAR**, **BCL** and **BAM** in the MMP-2 binding site are very similar and, thus, unsuitable to interpret the difference in term of binding affinities. In order to shed light on this experimental observation, MD simulations were carried out and free energy differences calculated with respect to the unsubstituted **BAR**. First, conventional MD simulations showed that the ligand stays in the binding pocket, as expected due to the bonded representation chosen for the Zn ion. The RMSD values of the protein and the ligand **BAR** remained below 0.4 nm throughout the simulation, indicating stability of the system on this timescale (Figure 8).

**Table 1:** GOLD fitness values for top-scored docking solutions.

ID	SCORE (kJ/mol)
**BAR**	69.72
**BCL**	70.79
**BAM**	71.02

**Figure 9:**
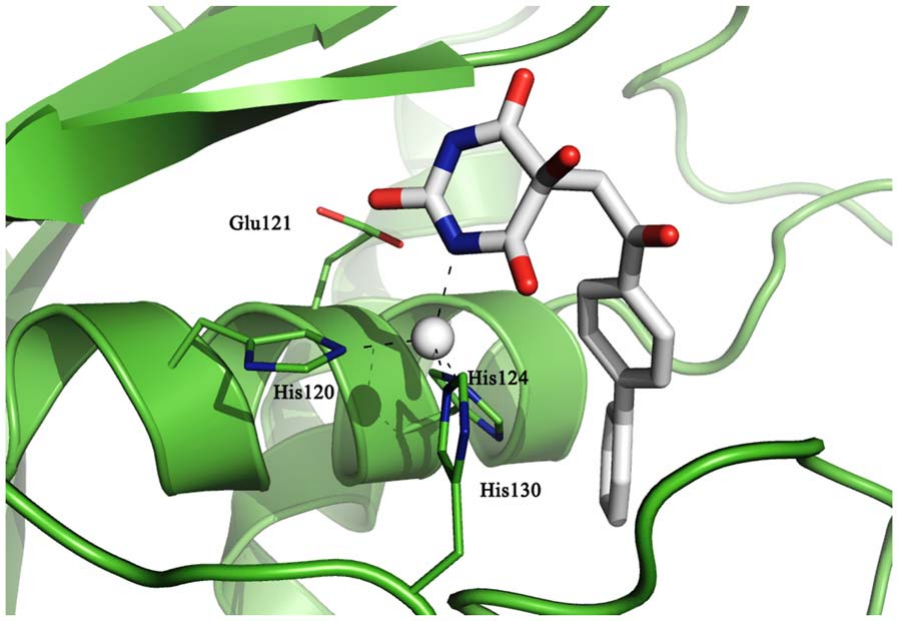
BAR interactions with the protein binding site. The **BAR** molecule (in white) contributes to the tetrahedral coordination of the catalytic Zn ion and establishes favourable interactions with the MMP-2 active site. Notice the hydrogen bond between the enolic hydroxyl of **BAR** and the charged Glu121 side chain. The biphenyl substituent is embedded into the protein hydrophobic pocket S_1_′.

As an example, Figure 9 shows **BAR** in the protein after 5 ns of simulation. The inhibitor contributed to the tetrahedral coordination of the catalytic Zn ion and was engaged in favourable interactions with the MMP-2 active site. In particular, a hydrogen bond occurred between the enolic hydroxyl of the inhibitor and the charged Glu121 side chain. As expected, the biphenyl substituent was embedded into the protein hydrophobic pocket S_1_′.

This structure was used as starting point for TI simulations of the transitions described above (i.e., **BAR** to **BCL** and **BAR** to **BAM**) and the backward transitions (i.e., **BCL** to **BAR** and **BAM** to **BAR**). Table 2 presents the free energy differences obtained by TI, along with the inhibition data. To assess the accuracy of our calculations, the results were compared with experimental binding data expressed as 50% Inhibitory Concentration (IC_50_) values (Figure 1). Experimental binding free energies were calculated as follows:
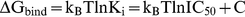
(3)where
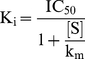
(4)is the Cheng-Prusoff equation to convert an IC_50_ value into an inhibition constant [48]. Since the applied computational procedure calculates only free energy differences, the value of the constant C becomes irrelevant.

**Table 2:** Calculated ΔΔG values of the forward and backward transitions of **BAR-BCL** and **BAR-BAM**.

Transition	ΔΔG_calc_ (forward)	ΔΔG_calc_ (backward)	ΔΔG_exp_
**BAR-BCL**	13.51±2.56	1.33±1.96	3.45
**BAR-BAM**	1.12±2.37	0.57±2.30	−0.446

Experimental ΔΔG values are also reported for comparison.

As reported in Table 2, free energy differences calculated via TI match the trend observed in the experimental data of binding affinity: as far as forward transitions are concerned, we might conclude that **BAR** and **BAM** have comparable binding free energies, while **BCL** corresponds to a pronounced low affinity. The error bars on these calculations are in the range of 2 kcal/mol. This is due to the extreme difficulty of obtaining stable values of dV/dλ for the case in which the molecule is bound to the protein. For consistency, we checked the results by performing the same calculations in the backward direction (i.e. **BCL** to **BAR** and **BAM** to **BAR**). It is evident in Table 3 that our calculations were fully consistent in water, even better than expected based on our error estimate. However the situation is dramatically different in presence of the protein, and, although a certain consistency might still be found in the case of **BAM**, there is certainly a significant discrepancy in the case of **BCL**. Increasing the time of the simulations by a factor of 2 did not contribute significantly to reducing the error bars, and the same calculation (in particular step 2 for the **BCL** case) performed with or without the SHAKE algorithm resulted in comparable values (data not shown). It is indeed clear from inspection of the data in Table 3 that our procedure is in trouble only in presence of the protein. Whether this is due to a poor sampling of protein conformations might be an issue for subsequent studies, but is certainly beyond the reach of the TI method. In order to clarify the source of this discrepancy, we decided to delve into further analysis of the equilibrium MD trajectories.

**Table 3:** Calculated ΔΔG values for the single steps considered in the forward and backward transitions of **BAR-BCL** and **BAR-BAM**.

Hydration	step1	err1	step2	err2	step3	err3	Total	Err
**BAR-BAM**	−6.24	0.41	3.69	1.24	−44.90	1.14	−47.45	1.74
**BAM-BAR**	45.40	1.15	−3.78	1.18	6.28	0.41	47.90	1.70
**BAR-BCL**	−5.99	0.41	6.76	0.91	−3.28	0.24	−2.51	1.03
**BCL-BAR**	3.29	0.25	−6.60	1.51	5.95	0.41	2.64	1.59

Complex	step1	err1	step2	err2	step3	err3	Total	Err
**BAR-BAM**	−6.24	0.33	1.43	1.23	−41.52	0.99	−46.33	1.61
**BAM-BAR**	42.48	0.91	−0.36	1.21	6.35	0.31	48.47	1.55
**BAR-BCL**	−5.45	0.27	19.79	2.32	−3.34	0.18	11.00	2.34
**BCL-BAR**	3.92	0.20	−6.70	1.08	6.75	0.29	3.98	1.14

Complex-water	step1	err1	step2	err2	step3	err3	Total	Err
**BAR-BAM**	0.00	0.53	−2.26	1.74	3.38	1.51	1.12	2.37
**BAM-BAR**	−2.92	1.47	3.42	1.69	0.08	0.51	0.57	2.30
**BAR-BCL**	0.54	0.49	13.03	2.49	−0.06	0.30	13.51	2.56
**BCL-BAR**	0.63	0.32	−0.10	1.86	0.80	0.50	1.33	1.95

**Figure 10:**
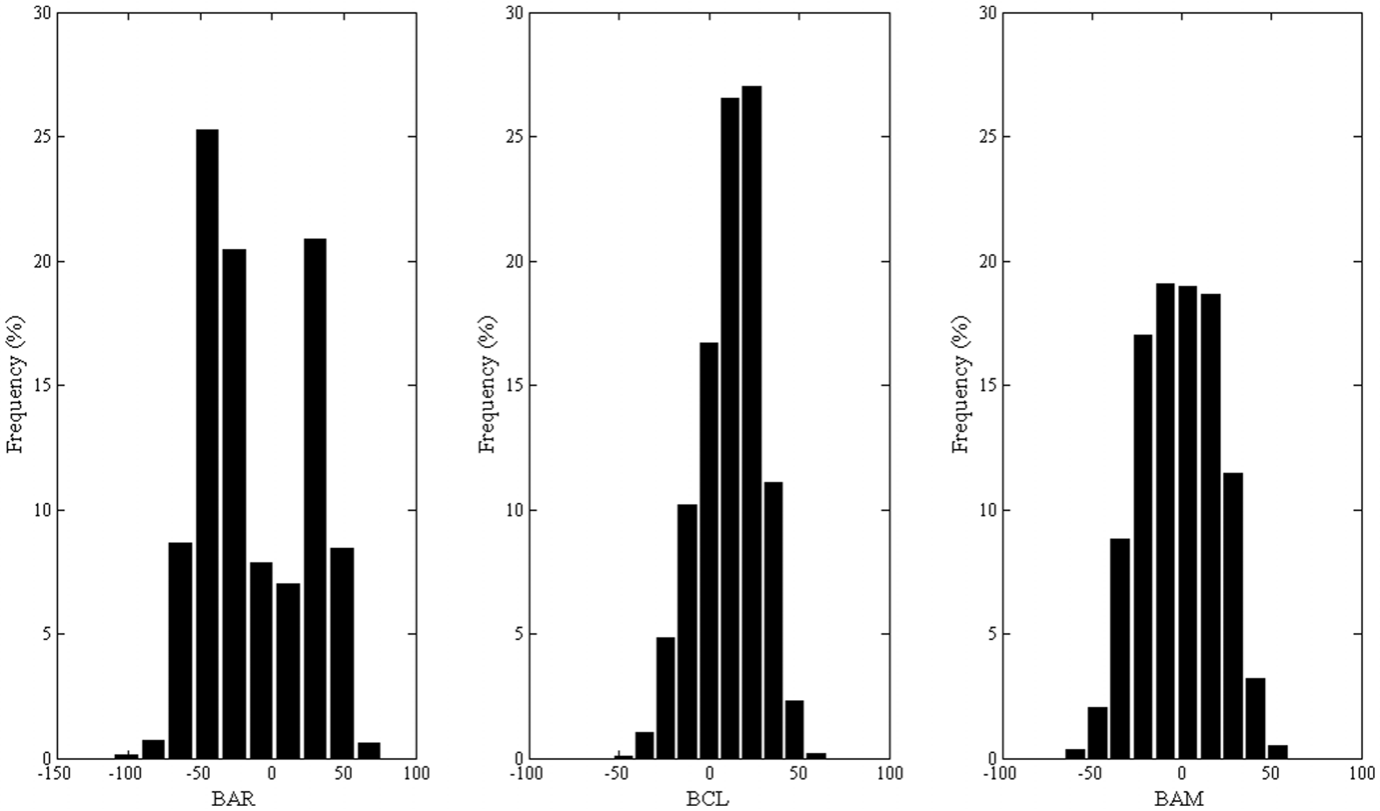
Histograms of the dihedral angles between the biphenyl rings. Distributions were obtained within 5 ns of equilibration MD trajectories. While the distribution for **BAR** is a symmetric bimodal centered on the expected values of ±45°, the one corresponding to **BAM** and **BCL** are peaked around the value 0°, hence pointing at a preferred planar conformation imposed by interaction with the binding pocket.

We report in Figure 10 the histograms of the values of the dihedral angle between the biphenyl rings for the three compounds examined.

**Figure 11:**
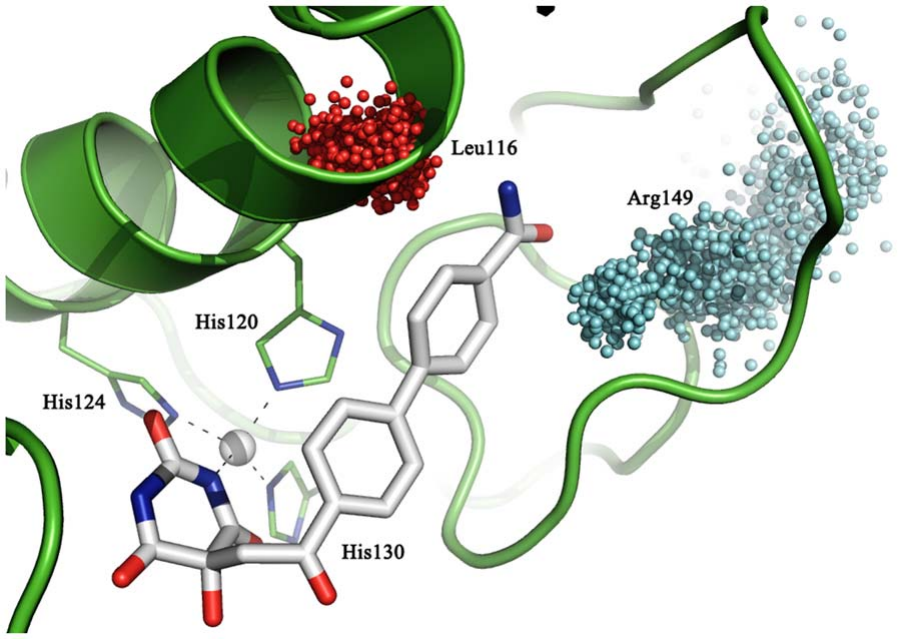
Interactions of BAM with the binding pocket. The final snapshot of the 5 ns MD trajectory is rendered in white capped stick models. Fitting spheres represent the positions visited during the 5 ns MD by the guanidine carbon atom of Arg149, colored in cyan, and by the oxygen backbone atom of Leu116, colored in red, of MMP-2 (PDB code 1HOV). Black dashed lines show the coordination to the catalytic zinc ion.

It is known that the dihedral angle between the biphenyl rings is about ±44°: this value was typically found in the case of **BAR**. It is evident that the biphenyl rings of **BAR** were preferably rotated to determine a bimodal distribution of the torsion angle approximately equal to ±45°. In the case of **BAM**, the distribution is no longer a bimodal centered on the two expected values, but rather a flattened Gaussian centered on the value 0°. The expected values of the dihedral angles are still visited within the time frame of our equilibration MD trajectory, although they are not sufficiently populated as in the case of **BAR**. This effect becomes dramatic in the case of **BCL**, for which the distribution of the dihedral angle becomes quite sharp and centered around 0°: hence, the introduction of a chlorine atom had the effect of forcing the biphenyl rings into a coplanar conformation supposedly better fitting into the S_1_′ pocket. A visual inspection, in fact, revealed that the chlorine atom is engaged in stable vdW interactions with Leu116, Val117 and Tyr142 residues which are all within 5 Å of the chlorine atom in all frames within the time scale of the MD simulations. We can conclude that a *meta* substitution on the second phenyl ring causes a steric hindrance such that the chlorine atom is blocked to remain in a hydrophobic pocket where it establishes favourable interactions with the protein residues, although this implies a reduced conformational freedom of the biphenyl fragment into the S_1_′ subsite. Diversely, a *para* substitution seems to be more tolerated, since it allows larger flexibility of the two phenyl rings although less than in **BAR** since the carboxamido group is involved in HB interactions with neighbouring residues. Specifically, it has been observed that the N atom of the carboxamido group interacts with the backbone of Leu116 and Asn147 and with the side chain of Thr145, while the O atom of the carboxamido group is able to contact the side chains of Tyr144 and Arg149 residues. Additionally, both atoms are engaged in HB interactions with water molecules in the pocket. The most frequent interactions are those established between the N atom of carboxamido group and carbonylic oxygen atom of Leu116, and between the O atom of the carboxamido group and the side chain of Arg149. Figure 11 shows the coordinates of the atoms involved in such interactions sampled over the whole trajectory. The HB with the Leu116 main chain is stable within the time scale of the MD simulation, while that with the Arg149 swings among the three N atoms of its side chain.

How the reported differences contributed to the accuracy of our free energy calculations can therefore be ascribed to the choice of the initial conformation and sampling issues. The initial conformation in the case of **BAR**, was slightly tilted and the distribution of the angles became close to that of **BAM** and **BCL** for high λ values, as expected. It is then evident that both compounds will tend, within the time frame of the calculation, to align the biphenyl rings. The calculation in the forward direction is therefore quite reliable, while we suspect that the one in the backward direction is affected by a poor sampling of the distribution of the angle between the biphenyl rings in **BAR**. In this respect, we would conclude that the backward calculation, at least in the case of **BCL**, should be completely disregarded, although it certainly represents an interesting case for subsequent more detailed calculations. In particular, in case of calculations involving a choice of reaction coordinates (or collective variables), our study points out the need to insert the angle between the biphenyl rings into the list of the variables to be taken into account.

In conclusion, the present study has shown that the alloxan core represents a novel ZBG ensuring a stable coordination to the catalytic zinc ion. Such a scaffold was actually employed as chemical intermediate for the preparation of pyrimidopyridazine compounds acting as inhibitors of MAO-B and MAO-A enzymes for the treatment of Parkinson's disease [19]. The alloxan core was selected via substructure search from our academic library containing a drug-like collection of molecular types designed to bias several therapeutically relevant enzymes. The bioactive potential of the alloxan core was thus successfully enlarged to the MMP-2, a class of enzymes also known as gelatinases that have a definite role in the breakdown of the extracellular matrix and in the turnover of collagen, a fundamental component of the basement membrane. The initial activities observed towards MMP-2 were significantly increased in the nanomolar range by the insertion of a biphenyl fragment, a privileged structure engaging the S_1_′ subsite. A value of pIC_50_ equal to 6.74 was found for **BAR** towards MMP-2. The need to optimize further the activity prompted us to rationally decorate the biphenyl ring. In this respect, the *para* position was functionalized with the carboxamido group to obtain **BAM**, the highest active compound with a pIC_50_ equal to 7.06. On the other hand, **BCL** was instead the derivative having a chlorine atom at the *meta* position of the biphenyl which determined a drop of activity with pIC_50_ being as low as 4.22. We have carried out MD and free energy calculations to investigate the binding of **BAR**, **BCL** and **BAM** to MMP-2. Basically, the higher activity of **BAM** is due to the occurrence of a hydrogen bonding network established between the carboxamido group buried into the deeper area of S_1_′ subsite with a number of polar residues of MMP-2. Although unable to dive into such polar zone, **BAR** is still able to fit the S_1_′ subsite with the result of preserving its activity. The weaker activity of **BCL** can be instead explained observing the diverse orientation of the biphenyl ring that is forced in a coplanar conformation due to the chlorine atom trapped in hydrophobic contacts with some hydrophobic residues. Notably, the alloxan-based compounds demonstrated activity towards the other gelatinase MMP-9 and in particular a good selectivity was observed for **BAM** being more than 20 fold selective towards MMP-2.

The basic idea behind our current investigation was the development of easy-to-prepare druglike molecules holding a stable tetrahedral coordination to the catalytic zinc ion while properly fit the S_1_′ specificity pocket of MMP-2. In this respect, we have explored the potential of alloxan derivatives and obtained the molecular rationale accounting for their different affinities. However, the design of novel MMP-2 inhibitors is still a fascinating challenge: for instance, an even more promising route in the direction of an enhanced molecular selectivity would target substrate binding exosites outside the active site. The computational strategy employed in this work might well represent a standard protocol in a further search for MMP-2 inhibitors.
